# Geographic Variation of Failure-to-Rescue in Public Acute Hospitals in New South Wales, Australia

**DOI:** 10.1371/journal.pone.0109807

**Published:** 2014-10-13

**Authors:** Hassan Assareh, Lixin Ou, Jack Chen, Kenneth Hillman, Arthas Flabouris, Stephanie J. Hollis

**Affiliations:** 1 Simpson Centre for Health Services Research, Australian Institute of Health Innovation & South Western Sydney Clinical School, University of New South Wales, Sydney, New South Wales, Australia; 2 Epidemiology, Western Sydney Local Health District, Sydney, New South Wales, Australia; 3 Intensive Care Unit, Royal Adelaide Hospital, Adelaide, South Australia, Australia; Emory University Rollins School of Public Health, United States of America

## Abstract

Despite the wide acceptance of Failure-to-Rescue (FTR) as a patient safety indicator (defined as the deaths among surgical patients with treatable complications), no study has explored the geographic variation of FTR in a large health jurisdiction. Our study aimed to explore the spatiotemporal variations of FTR rates across New South Wales (NSW), Australia. We conducted a population-based study using all admitted surgical patients in public acute hospitals during 2002–2009 in NSW, Australia. We developed a spatiotemporal Poisson model using Integrated Nested Laplace Approximation (INLA) methods in a Bayesian framework to obtain area-specific adjusted relative risk. Local Government Area (LGA) was chosen as the areal unit. LGA-aggregated covariates included age, gender, socio-economic and remoteness index scores, distance between patient residential postcode and the treating hospital, and a quadratic time trend. We studied 4,285,494 elective surgical admissions in 82 acute public hospitals over eight years in NSW. Around 14% of patients who developed at least one of the six FTR-related complications (58,590) died during hospitalization. Of 153 LGAs, patients who lived in 31 LGAs, accommodating 48% of NSW patients at risk, were exposed to an excessive adjusted FTR risk (10% to 50%) compared to the state-average. They were mostly located in state's centre and western Sydney. Thirty LGAs with a lower adjusted FTR risk (10% to 30%), accommodating 8% of patients at risk, were mostly found in the southern parts of NSW and Sydney east and south. There were significant spatiotemporal variations of FTR rates across NSW over an eight-year span. Areas identified with significantly high and low FTR risks provide potential opportunities for policy-makers, clinicians and researchers to learn from the success or failure of adopting the best care for surgical patients and build a self-learning organisation and health system.

## Introduction

Adverse events during hospitalisation and complications after surgery can be quite common. In the U.S., approximately one-fifth of patients who underwent surgery in 1997 died due to treatable complications [1]. In Australia, between 15 to 20% of patients experienced at least one complication after the surgery; 5% to 7% of them died prior to discharge [2]–[4]. Timely recognition and effective treatment of the complication once it occurs can prevent patient death [5]. Silber and colleagues [6] proposed to measure Failure-to-Rescue (FTR), defined as the proportion of deaths among surgical in-patients with treatable complications, as a hospital quality indicator. Effective management of treatable post-operative complications reportedly made a larger contribution to patient survival than pre-operation patient characteristics or operation type [7]. Since the Agency for Healthcare Research and Quality (AHRQ) adopted FTR as one of its patient safety indices [8], the concept has gained wide acceptance and now used to evaluate and compare post-operative care across hospitals [9]–[14]. FTR was found to have the highest incidence rate (91.13 per 1000 cases) among all the patient safety indicators and accounted for 6.7% of the total number of preventable patient safety incidents in the U.S. between 2007–2009 [15]. Two studies reported a decreased FTR rate of 2.4% to 6% within one decade [16], [17].

FTR rate varies across patients and hospitals with different characteristics. Older patients and those with higher pre-operative comorbidities have a higher risk of complication and risk of death after surgery [7], [18], [19]. Studies in both U.S. and European hospitals revealed that patients undergoing surgery in hospitals with a high mortality rate did not suffer from excessive complications compared to patients in lower mortality rate hospitals, but were less likely to survive due to lower quality of care [20]–[24]. Ghaferi and colleagues [25] also showed that patients in small hospitals which had only slightly higher complications rates (odds ratio 1.17), were exposed to substantially higher FTR rates compared to patients in large hospitals. They suggested that large hospitals were more capable of effectively rescuing patients from complications compared to their low volume counterparts.

The success of regional policy interventions and quality improvement programs varies, resulting in a different level of care quality across catchment areas. There are significant variations in post non-cardiac surgery mortality rates among 28 European nations [26] and in complication and mortality rates among U.S. hospitalised patients across different states during 2009–2011 [27], reflecting the potential different areal risk of FTR and other adverse outcomes. Furthermore, a better understanding of the spatial distributions of FTR may facilitate the design and prioritisation of regional quality improvement interventions [26].

Accordingly, we aimed to investigate the spatiotemporal pattern of post-surgery FTR rate for all public acute hospital patients in New South Wales (NSW), Australia during 2002–2009, in order to enhance our understanding of geographical variation of FTR across NSW.

## Methods

### Data source and study population

We used records from the NSW Admitted Patient Data Collection (APDC) database. The APDC is administrated by NSW Ministry of Health including all admitted patient services provided by NSW public and private healthcare facilities. The APDC includes information on patient demographics, medical conditions and procedures, hospital characteristics, and separations (discharges, transfers and deaths) from all public and private hospitals, as well as day procedure centres in NSW. The medical records for each episode of care in the APDC were assigned with codes based on the International Statistical Classification of Diseases and Related Health Problems, Tenth Revision, Australian Modification (ICD-10-AM) 4^th^ edition [28]. Of all admissions at 497 healthcare facilities in NSW between 1^st^ January 2002 to 31^st^ December 2009, we included all 82 public acute hospitals in NSW (9,221,128 admissions; 57.4% of all admissions) in our study, excluding community and private facilities, multipurpose and non-acute centres, psychiatric and rehabilitation facilities, nursing home and hospices, and two children's hospitals and one other hospital (data was unavailable).

### Measures and covariates

Following methodology by AHRQ, we defined FTR as mortality among surgical patients who developed at least one of six serious treatable complications during hospitalisation; including acute renal failure, deep vein thrombosis or pulmonary embolism, pneumonia, sepsis, shock or cardiac arrest, gastrointestinal haemorrhage or acute ulcer [8]. The six abovementioned complications were identified by secondary diagnostic codes (ICD-10-AM) that were translated from the AHRQ definition (ICD-9-CM) by Victorian Government Health Information [29]. Applying AHRQ inclusion criteria [8], patients who had elective surgery within two days of admission, aged between 18–90 years (inclusive) and who were transferred to an acute care facility were considered in the population study. 4,362,624 admissions in 82 acute hospitals were included (47.3% of all NSW public acute hospital admissions). Ethical approval was obtained from the University of NSW Human Research Ethics Committee (LNR/11/CIPHS/64).

We selected NSW Local Government Areas (LGA; 153 in total) as the spatial units through the aggregation of patient postcode (651 in total) using appropriate concordance references [30]. NSW is also divided into 15 Local Health Districts (LHDs) and three hospital networks which can be categorized into two classes: Metropolitan (eight LHDs and three networks), and Rural and Regional NSW (seven LHDs). Aggregated age and gender of patients and the distance between patient residential postcode and the treating hospital in each study year at LGA level were included in the adjustment. We also utilised a LGA-level advantage and disadvantage index of Socio-Economic Indices for Areas (SEIFA) as a covariate with lower values indicating more disadvantage in the area [31]. Similarly, using Accessibility/Remoteness Index Australia Plus (ARIA+), a remoteness score (varying between zero to fifteen with higher values indicating more remoteness) was obtained for each LGA and employed as a covariate [32]. Aggregated covariates was generated using mean for scale variables (age, SEIFA, ARIA+ and distance), and percentage of females (0–100) among all patients for gender. 77,130 admissions (%1.7 of 4,362,624 admissions) were excluded due to either missing items in covariates or patient's non-NSW residential location. We initially examined the Elixhauser and the Charlson Index comorbidities based on the ICD-10 coding scheme [33]. We did not include either of them in the adjustment given the recent reports that these indices may introduce misleading results possibly due to geographical variations and biases in the coding [34]–[36].

### Statistical analysis

To test the presence of any geographical variations of FTR across NSW LGAs over the study period (2002–2009), we examined spatial autocorrelation of the outcome across LGAs. We applied an empirical Bayes index modification of Moran's I [37] to evaluate the global association. Local spatial association measures (LISA) were also used to identify clusters. A cluster of neighbouring LGAs with high (or low) FTR rates and significant local indicators was regarded as a hot (or cold) spot [38], [39]. The neighbourhood effect for each LGA was limited to the first order of neighbours, LGAs with a shared common boundary, which implies that the neighbour LGAs are more related than distant LGAs. We then utilised spatiotemporal modelling techniques to investigate spatial and temporal patterns of adjusted FTR rates across LGAs during the study period. We derived areal adjusted relative risk (compared to overall FTR rate) using a geo-additive mixed effect model where a Poisson distribution underlies the observed FTR count, 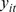, for 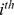 LGA, 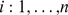, at 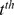 year, 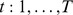 [40]. A Bayesian hierarchical model was defined to incorporate effects of covariates and spatial and temporal structure through random and fixed components. We followed the model specification proposed for spatiotemporal models in Gaussian Markov Random Field framework [41], [42]. For spatial patterns, we used two latent random effects components: a spatially structured random effect, 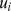, which was modelled as a so-called intrinsic Conditionally Autoregressive (iCAR); and a spatially unstructured effect, 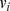, which was modelled as an independent normal distribution [41], [43]. The former accounts for any spatial autocorrelation, whilst the latter captures unexplained variation across LGAs and over dispersion. 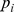 is the known LGA-specific offset parameter, the number of patients who developed complications. To model temporal patterns, we extended the parametric formulation proposed by Bernardinelli and colleagues [44]. In a semi-parametric approach, we included two time trends, linear and quadratic, as the fixed effect in the model (a linear, 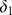, and a quadratic, 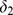), and then added a random effect term, 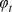, to account for any departure from the parametric fixed effects. We set time at the centre of study period (2005.5); therefore time varies between -3.5 and 3.5 for the linear term, and between 0.25 and 12.25 for the quadratic term. To incorporate interaction between space (LGAs) and time (linear and quadratic), two additional random effect terms, 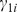 and 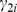, were incorporated into the model. These terms are analogous to the random slope formulation capturing model differential trends of the 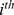 LGA, since they can be interpreted as the amount by which the time trends of *i^th^* LGA differ from the overall linear and quadratic time trends [44], [45]. Five year×LGA-specific covariates, 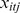, comprising average age, proportion of females in the population at risk, square root of average distance between patients residential postcode and treating hospital, SEIFA and ARIA+ were added into the model. Covariates were set as continues variables and assumed to have fixed effects.
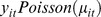





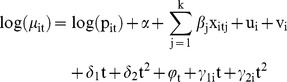



In prior specification, we let fixed effects, 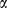, 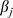, 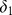 and 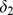, follow a zero-mean normal distribution with a very large variance of 10^3^. The unstructured random spatial term 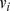, and all random effect terms, 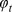, 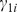 and 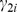, were set as a zero-mean normal distribution with an unknown precision following a Gamma distribution with shape and scale parameters of 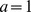 and 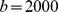, respectively. A similar Gamma prior was also used for the precision parameter of the structured spatial term, 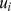, in iCAR. This setting led to non-informative priors with large variances imposing no pattern [46], [47]. To test if spatial effect is evident, we applied a recommended posterior-based probability, 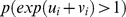, criterion with a 10% threshold [44], [48], [49]. To be consistent, a similar significance level was applied for other effects and analyses.

We employed non-informative zero-mean normal priors using the default and recommended settings for the precision [49]. Although prior specification in Bayesian setting is arbitrary and may lead to a different conclusion, this full Bayesian geo-additive model was reported to be relatively insensitive to the choice of prior [46]. In our sensitivity analysis, no deviation in results was seen when less defuse priors including a normal distribution with a variance of 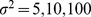 and a Gamma with a smaller scale parameter of 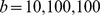 were employed. The robustness of the results was not unexpected due to the small effect sizes which were obtained for all components including intercept, 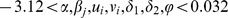. Moreover, use of a highly informative prior which may affect likelihood and lead to different results is not justifiable in the absence of any prior evidence. We limited the neighbouring effect to the LGAs with shared borders; other settings such as distance based configuration may be of interest for further investigation. The model without LGA-specific temporal patterns was preferred over the model with random space-time interaction terms, 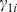 and 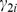, according to the smaller Deviance Information Criterion [50], 3064.8 vs. 3068.5, respectively. We only presented results from the superior model.

The spatiotemporal model was computed using Integrated Nested Laplace Approximation (INLA) methods. This is a computation technique for Bayesian latent Gaussian models which outperforms traditional Markov chain Monte Carlo (MCMC) methods while providing very precise estimates [42], [45], [51]. Data preparation was conducted in Stata 12.0 [52]. Statistical analyses and modelling were performed using INLA package [49], [53], built on 26 May 2013 (www.rinla.org) within R environment version 3.0.0 [54].

## Results

Of 4,285,494 elective surgical admissions for NSW residents during 2002–2009, across 82 NSW public acute hospitals, 58,590 (1.3%) patients residing in NSW developed at least one of the six complications of interest. Approximately 14% of them died prior to discharge, equivalent to an incidence rate of 1.88 per 1000 surgical patients. Males and older patients tended to have a higher risk of death compared to their counterparts (Table 1). Crude FTR rate slightly varied across years. There were large variations among LHDs (SD = 2.58%) and LGAs (SD = 4.28%). Patients who resided in metropolitan areas had lower crude FTR rates compared to those patients residing in non-metropolitan areas. Patients residing in regions with the highest SEIFA score (4^th^ quartile) had the lowest FTR rate. The remoteness of patients' residential locations and the distance between the residential postcode and the treating hospital were associated with different crude FTR rates. Patients residing in very remote areas had the highest FTR rate.

**Table 1:** Distribution and proportion of patients who developed at least one post-surgery complication (population at risk) and died (failure-to-rescue) across patients' and incidences' characteristics.

Characteristics		Patients at risk		Patients died		FTR rate
**Age** *						
	> = 18 yr&<35 yr	3602	(6.15%)	167	(2.03%)	4.64%
	> = 35 yr&<55 yr	9989	(17.05%)	719	(8.73%)	7.20%
	> = 55 yr&<75 yr	22564	(38.51%)	2974	(36.11%)	13.18%
	> = 75 yr<90	22435	(38.29%)	4376	(53.13%)	19.51%
	Mean ± SD^†^	66.23±16.61		72.22±13.44		-
	Median ± IQ^†^	70.00±23.18		75.84±17.01		-
**Gender** *						
	Female	27020	(46.12%)	3490	(42.37%)	12.92%
	Male	31570	(53.88%)	4746	(57.63%)	15.03%
	Mean ± SD (%Female)^†^	44.51%±6.86%		41.46%±13.60%		-
	Median ± IQ (%Female)^†^	45.42%±6.41%		42.22%±14.07%		-
**Quartiles of distance travelled** *						
	1^st^ quartile (<17.6 km)	32690	(55.79%)	4551	(55.25%)	13.92%
	2^nd^ quartile (> = 17.6 km & <71.1 km)	16418	(28.02%)	2458	(29.85%)	14.97%
	3^rd^ quartile (> = 71.1 km & <121.7 km)	6933	(11.83%)	858	(10.42%)	12.38%
	4^th^ quartile (> = 121.7 km)	2549	(4.36%)	369	(4.48%)	14.46%
	Mean ± SD^†^	88.22±88.42		97.76±97.44		-
	Median ± IQ^†^	71.11±104.04		83.10±118.83		-
**Year** *						
	2002	5402	(9.23%)	680	(8.25%)	12.59%
	2003	5116	(8.73%)	691	(8.39%)	13.51%
	2004	6371	(10.87%)	923	(11.21%)	14.49%
	2005	7085	(12.09%)	1023	(12.42%)	14.44%
	2006	7447	(12.71%)	1146	(13.91%)	15.39%
	2007	8560	(14.61%)	1222	(14.84%)	14.28%
	2008	9090	(15.51%)	1330	(16.15%)	14.63%
	2009	9519	(16.25%)	1221	(14.83%)	12.83%
	Mean ± SD^§^	7323.75±1648.05		1029.50±246.73		13.95%±0.95%
	Median ± IQ^§^	7226.00±2564.00		1085.00±355.80		14.30%±1.19%
**Quartiles of SEIFA** *						
	1^st^ quartile (most disadvantaged)	7883	(13.45%)	1215	(14.82%)	15.41%
	2^nd^ quartile	13911	(23.74%)	1877	(22.90%)	13.49%
	3^rd^ quartile	20857	(35.61%)	3219	(39.26%)	15.43%
	4^th^ quartile (most advantaged)	15939	(27.20%)	1887	(23.02%)	11.84%
**Categories of ARIA+** *						
	Highly Accessible (< = 0.2)	37194	(63.48%)	5345	(64.90%)	14.37%
	Accessible (>0.2 & < = 2.4)	13685	(23.36%)	1864	(22.63%)	13.62%
	Moderately Accessible (>2.4 & < = 5.92)	6862	(11.71%)	904	(10.97%)	13.17%
	Remote (>5.92 & < = 10.53)	607	(1.04%)	82	(1.00%)	13.53%
	Very Remote (>10.53 & < = 15)	242	(0.41%)	41	(0.50%)	16.83%
**Local health district** *						
	Metropolitan	35998	(61.44%)	4949	(60.09%)	13.75%
	Rural & Regional NSW	22592	(38.56%)	3287	(39.91%)	14.55%
	Mean ± SD^‡^	3661.85±2967.23		514.77±482.91		13.41%±2.58%
	Median ± IQ^‡^	2724.00±2395.00		410.20±280.20		12.54%±4.02%
**Local government areas**						
	Mean ± SD^†^	383.90±545.70		53.83±90.91		12.59%±4.28%
	Median ± IQ^†^	167.80±390.65		19.91±47.57		12.38%±5.21%
**Total**		58590		8236		14.06%

* Significant at 0.01 using χ2 test.

Note: 1502 FTR cases were excluded due to non-NSW postcodes. Summary statistics were calculated over (†) LGAs, (§) years 2002–2009, and (‡) LHDs.

**Figure 1:**
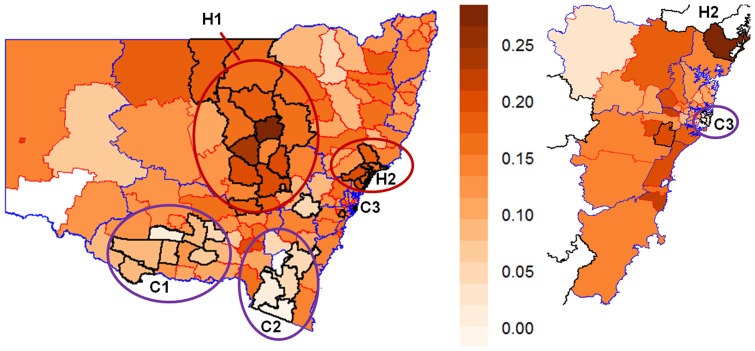
Crude FTR rate for each LGA (separated by red borders) in NSW over the study period (2002–2009). LGAs with significant LISA index (p-value<0.1), indication of clusters, are highlighted with black borders and labelled (H: hot spot, C: cold spot). LHDs are separated by blue borders. LGAs within Sydney metropolitan LHDs are enlarged in the right panel.

Figure 1 shows the overall crude rate of FTR for all LGAs in NSW within the study period (2002–2009). The most densely populated LGAs in NSW, located within Sydney Metropolitan LHDs are shown on the right panel of Figure 1. Wentworth LGA, the far south west region of NSW within Far West LHD, had no patients with a post-operative complication and, therefore, no FTR was observed. The rate varied from less than 1% in Bombala and Murrumbidgee LGAs both located in the two southern LHDs of NSW, to 26.6% in Gilgandra in the centre of the state within Western NSW LHD. A non-zero Moran I index of 0.325 (p-value = 0.001) obtained in the preliminary spatial analysis revealed a significant spatial autocorrelation. This positive value suggests neighbouring LGAs tended to have similar levels of FTR. Five main clusters each comprising of at least three highly correlated LGAs were identified based on LISA statistics. Two clusters with high FTR rates, H1 and H2 (hot spots), were located in the state's centre towards the northern part of NSW, largely within Western NSW LHD (H1), and in the eastern coastline of NSW within Hunter New England LHD (H2), with rate averages of 18.4% and 18.0%, respectively. Of three cold spots, clusters of LGAs with low rates, two were found in the southern part of NSW within Murrumbidgee (C1) and Southern NSW (C2) LHDs, and one in South Eastern Sydney LHD (C3), with average rates of 3.3%, 6.9%, and 6.7%, respectively. Clusters are presented in Figure 1.

**Figure 2:**
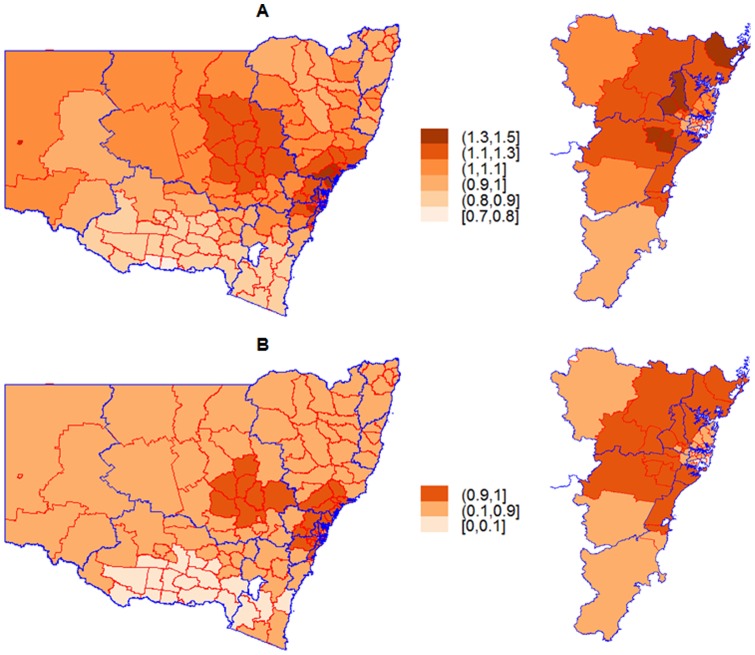
Smoothed relative risk of FTR for each LGA (separated by red borders) in NSW over the study period (2002–2009), adjusted for age, gender, distance, SEIFA and ARIA+ scores: (a) Relative risk posterior estimates (reference level is state average), 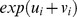; (b) Posterior probability of relative risk greater than 1 (state average), 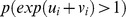. LHDs are separated by blue borders. LGAs within Sydney metropolitan LHDs are enlarged in the right panel.

Smoothed relative risks of FTR after adjustment for age, gender, time trend, distance, SEIFA and ARIA+ scores were obtained using a spatiotemporal model. Posterior estimates of rates are shown in Figure 2. Overall FTR rate in NSW was the reference level in the calculation of relative risks. LGAs with significant deviation from the state average are illustrated in Figure 2 by light or dark grey shading.

Of the 153 LGAs, nine regions had a significantly higher risk of FTR, with an excessive risk ranging from 30% to 50%, compared to the state average. These regions were mostly located in the mid-eastern coastline of NSW within Hunter New England, and Sydney Western and South Western LHDs, accommodating 24% of all patients at risk across NSW. Of 31 LGAs with a lower level of excessive risk (10% to 30%), 22 LGAs significantly deviated from the state average. They were found within Western NSW and Sydney Metropolitan LHDs. Overall, 48% of all NSW patients at risk resided within 31 LGAs with higher FTR rates; see Table S1 for more details. Eight LGAs had significantly lower FTR rate (RR ranged from 0.7 to 0.8), three in Sydney South Eastern LHD and five in the southern NSW. We also found 18 low FTR LGAs with a smaller deviation from the state average (RR ranged from 0.8 to 0.9), mostly within Murrumbidgee, Sydney and Sydney South Eastern LHDs. Areas with a lower risk of FTR accommodated 8% of NSW patients at risk; see Table S2 for more details.

**Figure 3:**
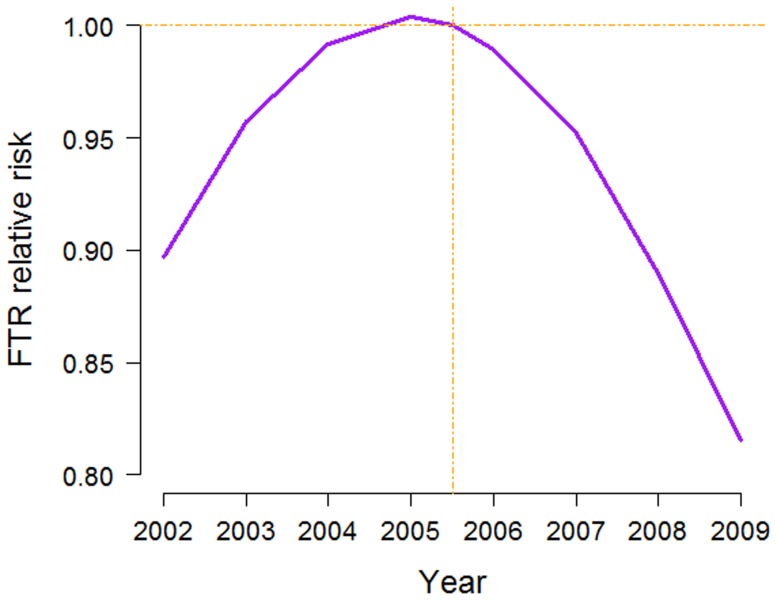
Temporal pattern of FTR in NSW over the study period (2002–2009). Relative risk posterior estimates were obtained from the spatiotemporal model, 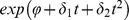, in which time was centred on mid-2005 and adjusted for age, gender, distance, SEIFA and ARIA+ scores.

We found a significant linear and quadratic time effect in the spatiotemporal model (Table 2), where the risk of FTR peaked between 2005 and 2006, and then declined afterwards (Figure 3). As summarised in Table 2, LGAs accommodating a greater proportion of older patients had a higher risk of FTR compared to regions with younger patients (RR = 1.03). LGAs with a higher percentage of female patients had a significantly lower FTR risk (RR = 0.97). Socio-economically advantaged LGAs exhibited a lower risk of FTR (RR = 0.99). Remoteness of patients' residence area was not associated with FTR. Patients who underwent surgery in farther hospitals experienced a higher risk of FTR (RR = 1.021).

**Table 2:** Relative risk posterior estimates and 90% credible intervals for temporal effect and covariates in the spatiotemporal model of FTR across LGAs of NSW over the study period (2002–2009).

Covariates	RR	90% CI
Year	0.986	0.976–0.996
Year∧2	0.987	0.982–0.991
Age	1.033	1.026–1.040
Gender (% female)	0.967	0.941–0.995
Distance travelled	1.021	1.000–1.042
SEIFA score	0.989	0.983–0.996
ARIA+ score	0.978	0.939–1.018

Note: Effects of year (linear and quadratic) were obtained where time was centred on the middle of study period (2005.5). Effect of SEIFA score was obtained for 10 unit increments in the raw scores varying from 816 to 1155 in NSW. Effect of distance travelled was obtained for square root of the raw values. Effect of gender was obtained for 10 unit increments in the percentage of females among all patients.

## Discussion

We simultaneously studied spatial and temporal variation of post-operative failure-to-rescue in 82 public acute hospitals across NSW between 2002 and 2009. We found 8236 (14%) deaths among patients who developed at least one complication after surgery. Patients residing in 31 LGAs mostly located in the centre and mid-eastern coast line of NSW were exposed to a higher risk of FTR (varying from 10% to 50%) compared to the state average. In contrast, within 26 LGAs, located in the southern part of NSW and Sydney eastern regions, levels of FTR risk were 10% to 30% lower. The yearly FTR rate was in the highest level in 2005.

Timely recognition and management of post-operative complications has been considered as a patient safety indicator [8], [29], [55]. Hospitals responding differently to such complications results in the inequality of care provided and varying post-complication death rate at the hospital level and different geographic regions. [15], [21], [26], [27]. Our study demonstrated such a variation across LGAs of NSW. It showed that despite the close proximity of some areas, in particular within Sydney LHDs where several hospitals are accessible, patients residing in some LGAs were exposed to a higher level of FTR risk than those residing in neighbouring regions. Variation in the practice of quality initiatives aiming at the effective recognition and response to surgical complications at hospital and regional levels, as well as differences in the hospital characteristics, may have contributed to the observed discrepancies. For example, one study showed that the implementation of a Medical Emergency Team (MET) system was associated with a significant decrease in complication and post-operative death rates [56], [57]. It has also been shown that large hospitals have a lower FTR rate compared to small hospitals [25]. However, our study found that the LGAs comprising of highly populated areas within metropolitan LHDs with large hospitals had excessive FTR risks. Blacktown (in the western Sydney area) and Newcastle (North of Sydney) areas, which accommodated over 10% of all patients at risk, exhibited excessive FTR risks above 43%. Conversely, patients from areas with a lower population density such as in western parts of NSW, who predominantly underwent surgery in small centres, did not experience a higher FTR rate. Further hospital-level investigation is required to address and verify the effect of volume, staffing and other centre-based characteristics [14], [58].

The overall FTR rate of 14% in NSW was close to the national rates seen in the U.S.; 15.3% to 10.3% between 1998 and 2007 [16]. During 2002–2009 in NSW, the current study confirmed the significant quadratic trend of FTR found in our previous study using individual patient data [59]. In contrast with the consistently decreasing trend in the U.S. [16], NSW demonstrated an increasing trend until 2005–2006, followed by a decreasing trend to 2009. More importantly, our study showed that such a recent decreasing FTR trend was uniform across all different LGAs. Such trajectory in the FTR rate may be due to national and state-wide quality and safety programs advocated by Australian Commission on Safety and Quality in Healthcare (ACQSHC) [60] and Clinical Excellence Commission (CEC) of NSW [61] founded in 2006 and late 2004, respectively. CEC and ACQSHC had launched and sustained specific programs in targeting specific complications such as deep vein thrombosis and sepsis.

Our finding that older patients experienced a higher risk of FTR is consistent with previous study findings [7], [18]. However, our findings that male patients had a higher rate of FTR was in line with one study [18] but not others which reported no gender effect on post-operative mortality [3], [62], [63]. Further analysis on patient characteristics at an individual level, not LGA-aggregated, is required for verification of gender effect or other potentially contributing factors.

Patient safety indicators have been introduced in the U.S. and Australia [8], [29] and currently the routine measurement and reporting of indicators have been broadly adopted [15], [20], [27]. To our knowledge, the current study was the first to investigate the geographical variation of FTR in a large health jurisdiction in Australia. Our findings provide policy-makers with FTR risk distribution that is not just patient or hospital based. It presents results on a large geographical scale that may reflect the impact of multi-factorial determinants such as local area health policy, quality improvement initiatives, hospital culture and compliance with best practice. It may also be related to complex patients-level factors (i.e. age, gender, case mix, severity and comorbidity). Moreover, other environmental factors such as transportation, local referral system and accessibility of different health facilities may also play a role in patient outcomes. We examined the effects of remoteness of patients' residence locations and distance between patient residential address and treating hospital. We found that the proximity to the treating hospital was associated with the reduced FTR rate. Despite that hospital location was reported as a contributing factor [17], [64], no study directly investigated the impact of the distance between patients residential location and the treating hospital. Our identified hot spots provide local health authorities and hospital networks with pre-targeted areas for in-depth case studies on how and why this was the case and what lessons could be learnt and shared. Equally, cold spots may provide decision-makers, clinicians and researchers with valuable insights into how post-surgical care could be better delivered and avoidable deaths prevented. Although this study did not primarily aim to directly identify and compare the best or worst performers, it illustrates areas in which preferred practices and outcomes are delivered. The presence of geographical variation across LGAs and LHDs also reinforces the importance of continual monitoring and public reporting of health system performances in order to create a self-learning health system with a focus on patient-centred care [65].

Finally, the current study utilised the most recent development in computation techniques from Bayesian hierarchical modelling framework for spatiotemporal modelling. This approach allowed us a stable and fast estimation of the most complex spatial regression model in comparison to the conventional MCMC approach [42], [45], [51]. Our study included all NSW public acute hospital patients spanning an eight–year period and was based on an accepted AHRQ definition which makes cross-country comparison possible. Our study however, had some limitations. Firstly, our model was constructed on a LGA level which may suffer from ecological fallacy. As a result, our model only included limited covariates in the adjustment. We did not specifically examine the relationship between hospital level outcome and geo-spatial distributions. Future studies, however, should investigate other extended models to explore the impact of patient and hospital characteristics on the outcome. These results would help to identify high risk patients prior to their operation and evaluate the effects of post-operative quality improvement policies and initiatives [20], [57].

## Conclusions

There were significant spatial-temporal variations of FTR across NSW over an eight-year span. Such variations demonstrate a significant ecological effect that cannot be explained purely by hospital differences. Both the hot spots and cold spots identified provide potential opportunities for policy-makers, clinicians and researchers to learn from the success or failure of adopting the best care for surgical patients in building a self-learning organisation and health system.

## Supporting Information

Table S1 **List of LGAs with a significantly higher adjusted relative risk of FTR.**
(DOCX)

Table S2 **List of LGAs with a significantly lower adjusted relative risk of FTR.**
(DOCX)
