# Supplementary material for: Geographic Variation of Failure-to-Rescue in Public Acute Hospitals in New South Wales, Australia
Source: PLoS One. 2014 Oct 13;9(10):e109807. doi: 10.1371/journal.pone.0109807 (PMC4195695; doi:10.1371/journal.pone.0109807)
Supplement: Table S1 — List of LGAs with a significantly higher adjusted relative risk of FTR. (DOCX) [file pone.0109807.s001.docx]

# Supporting information

Table S1. List of LGAs with a significantly higher adjusted relative risk of FTR (compared to the state average) of patients from public acute hospitals of NSW between 2002 and 2009.

| **Local government area** | **Local health**  **district (type)** | **Patients at risk (% of NSW)** | | **Observed FTR rate** | **Average of age** | **% of females** | **Distance travelled-km (Quartile)** | | **SEIFA score (Quartile)** | | **ARIA+ score (Quartile)** | | **Adjusted relative risk of FTR (P>1)** | |
| --- | --- | --- | --- | --- | --- | --- | --- | --- | --- | --- | --- | --- | --- | --- |
| Wyong | Central Coast (M) | 1366 | (2.33%) | 20.25% | 67.27 | 43.06% | 29.87 | (2) | 942 | (2) | 0.17 | (2) | 1.44 | (0.999) |
| Blacktown | Western Sydney (M) | 3963 | (6.76%) | 17.25% | 64.85 | 47.30% | 8.21 | (1) | 974 | (3) | 0.00 | (1) | 1.44 | (0.999) |
| Newcastle | Hunter New England (R) | 2044 | (3.49%) | 19.27% | 68.96 | 44.64% | 7.81 | (1) | 991 | (3) | 0.01 | (1) | 1.43 | (0.999) |
| Cessnock | Hunter New England (R) | 814 | (1.39%) | 19.69% | 65.65 | 46.12% | 34.32 | (2) | 922 | (1) | 0.97 | (2) | 1.42 | (0.999) |
| Lake Macquarie | Hunter New England (R) | 2687 | (4.59%) | 18.42% | 67.97 | 45.70% | 15.21 | (1) | 985 | (3) | 0.05 | (1) | 1.41 | (0.999) |
| Maitland | Hunter New England (R) | 840 | (1.43%) | 20.08% | 68.11 | 43.87% | 26.90 | (2) | 986 | (3) | 0.31 | (2) | 1.40 | (0.999) |
| Campbelltown | South Western Sydney (M) | 1418 | (2.42%) | 16.69% | 62.38 | 49.46% | 18.19 | (2) | 943 | (2) | 0.11 | (2) | 1.39 | (0.999) |
| Camden | South Western Sydney (M) | 391 | (0.67%) | 16.53% | 65.38 | 47.53% | 19.84 | (2) | 1056 | (4) | 0.18 | (2) | 1.36 | (0.998) |
| The Hills Shire | Western Sydney (M) | 943 | (1.61%) | 14.97% | 67.21 | 49.14% | 11.65 | (1) | 1128 | (4) | 0.03 | (1) | 1.34 | (0.999) |
| Hawkesbury | Nepean Blue Mountains (M) | 231 | (0.39%) | 15.64% | 60.28 | 40.33% | 43.06 | (2) | 1017 | (4) | 0.99 | (2) | 1.29 | (0.998) |
| Fairfield | South Western Sydney (M) | 1903 | (3.25%) | 17.81% | 66.43 | 47.12% | 7.96 | (1) | 886 | (1) | 0.00 | (1) | 1.29 | (0.999) |
| Gosford | Central Coast (M) | 1003 | (1.71%) | 14.32% | 66.67 | 41.91% | 27.30 | (2) | 1001 | (4) | 0.42 | (2) | 1.23 | (0.994) |
| Hornsby | Northern Sydney (M) | 858 | (1.46%) | 12.84% | 65.89 | 49.91% | 9.93 | (1) | 1106 | (4) | 0.06 | (1) | 1.22 | (0.980) |
| Port Stephens | Hunter New England (R) | 792 | (1.35%) | 15.74% | 66.78 | 45.13% | 40.74 | (2) | 970 | (3) | 0.65 | (2) | 1.21 | (0.991) |
| Gilgandra | Western NSW (R) | 67 | (0.11%) | 26.59% | 65.91 | 32.07% | 163.32 | (4) | 911 | (1) | 4.68 | (4) | 1.21 | (0.940) |
| Narromine | Western NSW (R) | 75 | (0.13%) | 24.49% | 64.01 | 43.56% | 149.97 | (4) | 922 | (1) | 4.21 | (4) | 1.21 | (0.954) |
| Parkes | Western NSW (R) | 168 | (0.29%) | 19.36% | 64.08 | 41.52% | 115.03 | (3) | 933 | (1) | 3.85 | (4) | 1.21 | (0.946) |
| Dungog | Hunter New England (R) | 109 | (0.19%) | 17.27% | 64.02 | 40.74% | 54.73 | (2) | 978 | (3) | 1.68 | (2) | 1.20 | (0.966) |
| Sutherland Shire | South Eastern Sydney (M) | 1286 | (2.19%) | 15.96% | 71.68 | 48.87% | 8.48 | (1) | 1083 | (4) | 0.06 | (1) | 1.19 | (0.984) |
| Penrith | Nepean Blue Mountains (M) | 966 | (1.65%) | 12.00% | 61.29 | 45.39% | 16.14 | (1) | 989 | (3) | 0.03 | (1) | 1.19 | (0.983) |
| Singleton | Hunter New England (R) | 380 | (0.65%) | 12.56% | 63.06 | 44.63% | 36.91 | (2) | 1007 | (4) | 2.10 | (2) | 1.19 | (0.982) |
| Shellharbour | Illawarra Shoalhaven (M) | 426 | (0.73%) | 15.02% | 64.25 | 45.45% | 36.96 | (2) | 961 | (3) | 0.20 | (2) | 1.18 | (0.933) |
| Wollondilly | South Western Sydney (M) | 395 | (0.67%) | 13.91% | 65.70 | 47.62% | 31.87 | (2) | 1033 | (4) | 0.47 | (2) | 1.17 | (0.974) |
| Wollongong | Illawarra Shoalhaven (M) | 1206 | (2.06%) | 16.42% | 70.39 | 45.55% | 24.65 | (2) | 981 | (3) | 0.15 | (2) | 1.17 | (0.979) |
| Wellington | Western NSW (R) | 114 | (0.19%) | 19.93% | 67.84 | 38.94% | 123.36 | (4) | 899 | (1) | 3.28 | (3) | 1.16 | (0.902) |
| Cabonne | Western NSW (R) | 164 | (0.28%) | 19.99% | 66.44 | 48.47% | 77.05 | (3) | 993 | (3) | 2.68 | (3) | 1.16 | (0.960) |
| Liverpool | South Western Sydney (M) | 1337 | (2.28%) | 12.68% | 63.69 | 45.61% | 9.09 | (1) | 968 | (3) | 0.01 | (1) | 1.16 | (0.973) |
| Blue Mountains | Nepean Blue Mountains (M) | 389 | (0.66%) | 11.57% | 59.94 | 45.76% | 36.22 | (2) | 1038 | (4) | 0.12 | (2) | 1.15 | (0.916) |
| Dubbo | Western NSW (R) | 388 | (0.66%) | 13.50% | 64.60 | 47.75% | 97.85 | (3) | 965 | (3) | 3.10 | (3) | 1.13 | (0.902) |
| Parramatta | Western Sydney (M) | 1429 | (2.44%) | 13.70% | 67.63 | 51.57% | 7.29 | (1) | 996 | (3) | 0.00 | (1) | 1.13 | (0.949) |
| Mid-Western Regional | Western NSW (R) | 289 | (0.49%) | 14.19% | 62.64 | 45.81% | 96.92 | (3) | 951 | (2) | 3.18 | (3) | 1.13 | (0.917) |

(M) and (R) are acronyms for “Metropolitan” and “Rural and Regional” LHDs. Relative risk of FTR was obtained through spatiotemporal model and adjusted for age, % of females and SEIFA score at LGA level.
