# Supplementary material for: Geographic Variation of Failure-to-Rescue in Public Acute Hospitals in New South Wales, Australia
Source: PLoS One. 2014 Oct 13;9(10):e109807. doi: 10.1371/journal.pone.0109807 (PMC4195695; doi:10.1371/journal.pone.0109807)
Supplement: Table S2 — List of LGAs with a significantly lower adjusted relative risk of FTR. (DOCX) [file pone.0109807.s002.docx]

# Supporting Information

Table S2. List of LGAs with a significantly lower adjusted relative risk of FTR (compared to the state average) of patients from public acute hospitals of NSW between 2002 and 2009.

| **Local government area** | **Local health**  **district (type)** | **Patients at risk (% of NSW)** | | **Observed FTR rate** | **Average of age** | **% of females** | **Distance travelled-km (Quartile)** | | **SEIFA score (Quartile)** | | **ARIA+ score (Quartile)** | | **Adjusted relative risk of FTR (P>1)** | |
| --- | --- | --- | --- | --- | --- | --- | --- | --- | --- | --- | --- | --- | --- | --- |
| Deniliquin | Murrumbidgee (R) | 100 | (0.17%) | 8.51% | 69.01 | 43.26% | 64.97 | (2) | 926 | (1) | 4.31 | (4) | 0.76 | (0.046) |
| Waverley | South Eastern Sydney (M) | 243 | (0.41%) | 6.59% | 65.88 | 48.51% | 5.48 | (1) | 1101 | (4) | 0.00 | (1) | 0.77 | (0.042) |
| Queanbeyan | Southern NSW (R) | 130 | (0.22%) | 5.64% | 61.47 | 47.28% | 53.09 | (2) | 1051 | (4) | 1.04 | (2) | 0.78 | (0.079) |
| Randwick | South Eastern Sydney (M) | 730 | (1.25%) | 7.59% | 64.92 | 50.82% | 4.45 | (1) | 1063 | (4) | 0.00 | (1) | 0.79 | (0.015) |
| Botany Bay | South Eastern Sydney (M) | 269 | (0.46%) | 6.65% | 65.76 | 43.26% | 4.63 | (1) | 985 | (3) | 0.00 | (1) | 0.79 | (0.021) |
| Corowa Shire | Murrumbidgee (R) | 108 | (0.18%) | 10.53% | 68.50 | 43.21% | 67.91 | (2) | 943 | (2) | 2.02 | (2) | 0.80 | (0.032) |
| Berrigan | Murrumbidgee (R) | 46 | (0.08%) | 13.26% | 74.56 | 45.24% | 114.02 | (3) | 938 | (2) | 2.79 | (3) | 0.80 | (0.048) |
| Albury | Network with Vic (R) | 340 | (0.58%) | 10.19% | 67.84 | 47.30% | 17.32 | (1) | 967 | (3) | 0.94 | (2) | 0.80 | (0.046) |
| Murray | Murrumbidgee (R) | 18 | (0.03%) | 8.05% | 66.83 | 46.34% | 64.28 | (2) | 967 | (3) | 4.23 | (4) | 0.80 | (0.067) |
| Greater Hume Shire | Murrumbidgee (R) | 71 | (0.12%) | 10.71% | 70.34 | 47.19% | 78.58 | (3) | 976 | (3) | 2.68 | (3) | 0.81 | (0.023) |
| Lockhart | Murrumbidgee (R) | 37 | (0.06%) | 6.21% | 65.73 | 34.44% | 81.80 | (3) | 983 | (3) | 2.95 | (3) | 0.81 | (0.044) |
| Jerilderie | Murrumbidgee (R) | 18 | (0.03%) | 8.48% | 65.54 | 40.63% | 131.54 | (4) | 983 | (3) | 4.09 | (4) | 0.81 | (0.049) |
| Snowy River | Southern NSW (R) | 35 | (0.06%) | 1.15% | 59.42 | 64.85% | 86.20 | (3) | 1031 | (4) | 3.30 | (3) | 0.81 | (0.060) |
| Conargo | Murrumbidgee (R) | 22 | (0.04%) | 8.48% | 68.89 | 43.36% | 67.44 | (2) | 1039 | (4) | 4.32 | (4) | 0.81 | (0.047) |
| Urana | Murrumbidgee (R) | 14 | (0.02%) | 12.60% | 67.74 | 22.95% | 108.80 | (3) | 925 | (1) | 3.56 | (3) | 0.82 | (0.039) |
| Cooma-Monaro | Southern NSW (R) | 76 | (0.13%) | 5.05% | 64.92 | 45.38% | 34.04 | (2) | 976 | (3) | 2.43 | (3) | 0.83 | (0.044) |
| Wagga Wagga | Murrumbidgee (R) | 414 | (0.71%) | 9.50% | 64.68 | 52.06% | 74.27 | (3) | 987 | (3) | 2.19 | (2) | 0.83 | (0.015) |
| Tumut Shire | Murrumbidgee (R) | 117 | (0.20%) | 10.19% | 67.09 | 44.98% | 109.26 | (3) | 936 | (2) | 2.64 | (3) | 0.84 | (0.045) |
| Murrumbidgee | Murrumbidgee (R) | 15 | (0.03%) | 0.73% | 63.01 | 27.15% | 186.03 | (4) | 919 | (1) | 4.65 | (4) | 0.84 | (0.050) |
| Tumbarumba | Murrumbidgee (R) | 35 | (0.06%) | 16.69% | 68.29 | 42.02% | 109.21 | (3) | 944 | (2) | 2.97 | (3) | 0.84 | (0.082) |
| Narrandera | Murrumbidgee (R) | 106 | (0.18%) | 9.36% | 65.71 | 32.20% | 124.66 | (4) | 912 | (1) | 3.27 | (3) | 0.84 | (0.032) |
| Junee | Murrumbidgee (R) | 85 | (0.14%) | 7.26% | 67.52 | 49.03% | 83.73 | (3) | 932 | (1) | 2.13 | (2) | 0.84 | (0.064) |
| Rockdale | South Eastern Sydney (M) | 706 | (1.21%) | 10.34% | 70.51 | 46.80% | 4.50 | (1) | 1001 | (4) | 0.00 | (1) | 0.85 | (0.038) |
| Coolamon | Murrumbidgee (R) | 39 | (0.07%) | 4.36% | 64.82 | 44.39% | 122.84 | (4) | 960 | (3) | 3.12 | (3) | 0.85 | (0.076) |
| Burwood | Sydney (M) | 470 | (0.80%) | 10.17% | 70.23 | 46.94% | 7.58 | (1) | 1022 | (4) | 0.00 | (1) | 0.86 | (0.066) |
| Eurobodalla | Southern NSW (R) | 334 | (0.57%) | 11.67% | 67.12 | 44.17% | 66.05 | (2) | 940 | (2) | 2.43 | (3) | 0.88 | (0.097) |

(M) and (R) are acronyms for “Metropolitan” and “Rural and Regional” LHDs. Relative risk of FTR was obtained through spatiotemporal model and adjusted for age, % of females and SEIFA score at LGA level
